# Exploring Self-Supervised Vision Transformers for Gait Recognition in the Wild

**DOI:** 10.3390/s23052680

**Published:** 2023-03-01

**Authors:** Adrian Cosma, Andy Catruna, Emilian Radoi

**Affiliations:** Faculty of Automatic Control and Computer Science, University Politehnica of Bucharest, 006042 Bucharest, Romania

**Keywords:** gait recognition, biometric authentication, vision transformer, pose estimation, self-supervised learning, contrastive learning

## Abstract

The manner of walking (gait) is a powerful biometric that is used as a unique fingerprinting method, allowing unobtrusive behavioral analytics to be performed at a distance without subject cooperation. As opposed to more traditional biometric authentication methods, gait analysis does not require explicit cooperation of the subject and can be performed in low-resolution settings, without requiring the subject’s face to be unobstructed/clearly visible. Most current approaches are developed in a controlled setting, with clean, gold-standard annotated data, which powered the development of neural architectures for recognition and classification. Only recently has gait analysis ventured into using more diverse, large-scale, and realistic datasets to pretrained networks in a self-supervised manner. Self-supervised training regime enables learning diverse and robust gait representations without expensive manual human annotations. Prompted by the ubiquitous use of the transformer model in all areas of deep learning, including computer vision, in this work, we explore the use of five different vision transformer architectures directly applied to self-supervised gait recognition. We adapt and pretrain the simple ViT, CaiT, CrossFormer, Token2Token, and TwinsSVT on two different large-scale gait datasets: GREW and DenseGait. We provide extensive results for zero-shot and fine-tuning on two benchmark gait recognition datasets, CASIA-B and FVG, and explore the relationship between the amount of spatial and temporal gait information used by the visual transformer. Our results show that in designing transformer models for processing motion, using a hierarchical approach (i.e., CrossFormer models) on finer-grained movement fairs comparatively better than previous whole-skeleton approaches.

## 1. Introduction

How we move contains significant clues about ourselves. In particular, our gait (manner of walking) has been closely studied in medicine [1], psychology [2], and sports science [3]. Recently, gait analysis has received increased attention [4,5] from the computer science community coinciding with the exponential progress of deep learning and widespread availability of computing hardware. AI-powered gait analysis systems have been able to successfully recognize subjects [6,7,8,9,10], estimate demographics such as gender and age [11], and estimate external attributes such as clothing [12], without using any external appearance cues. These results are not surprising, given the large amount of individual differences in gait, which are due to differences in musculoskeletal structure, genetic and environmental factors, as well as the walker’s emotional state and personality [13].

Current systems are only really trained and tested in controlled indoor environments. Most methods use the CASIA-B dataset [6] as the standard benchmark for gait recognition models, containing 124 subjects walking indoors in a strictly controlled manner captured with multiple cameras. Complexity in the real-world cannot be fully modeled by such restrained scenarios. Only recently the focus has been on modeling gait “in the wild”, with datasets such as DenseGait [12], GREW [7], and Gait3D [14].

Gathering a large-scale dataset that is clean and fully annotated represents a tremendous effort in terms of both financial resources and allocated time. The GREW dataset [7] reportedly took 3 months of continuous work to be gathered and annotated. While such approaches have been useful in developing neural architectures for processing gait [8,9], they are not sufficiently diverse to be properly used in more relaxed, real-world environments. The AI community has been slowly moving away from this approach in other areas as well, with methods for self-supervised learning for both vision [15] and language [16] gaining significant traction, and often surpassing traditional supervised methods. Recent progress in self-supervised learning showed that self-supervised models are more robust and exhibit emerging behaviors, not explicitly defined during training. For instance DINO [17], a vision transformer trained in a self-supervised regime, learned class-specific features enabling unsupervised object segmentation without using any such labels during training. Cosma and Radoi [10] proposed the first contrastive method for self-supervised learning for gait analysis, by training a ST-GCN [18] on a smaller version of DenseGait [12]. Their method obtained reasonable results on downstream gait recognition tasks and showed that there is a strong correlation between the pretrained dataset size and zero-shot transfer performance.

While many approaches for gait analysis have been utilizing silhouettes extracted from background subtraction [6,8,9], extracting silhouettes in real surveillance scenarios implies the use of more advanced techniques, such as instance segmentation [19], which come at a significant computational cost. Sequences of silhouettes occupy significant storage space and are not sufficiently flexible to be used in other adjacent tasks, such as activity recognition. Moreover, silhouettes encode subtle appearance cues, which makes it unclear to what extent movement is utilized in the identification [20]. On the other hand, 2D pose estimation models have become increasingly accurate and computationally efficient [21,22]. Skeletons are cheap to extract, currently more reliable than 3D meshes and 3D poses, especially at a distance. Moreover, 2D skeletons are significantly more lightweight than silhouettes in terms of long-term storage.

Current architectures for processing sequences of skeletons are utilizing the natural spatial graph structure present in the human skeleton, introducing an inductive bias in the model design. Models such as the popular ST-GCN [18] and MS-G3D [23] have seen impressive results for skeleton-based action recognition.

Concurrently, there has been an explosion in the use of transformer models in almost all areas of deep learning since their initial application for natural language processing. Transformers are considered a more general architecture, with few inductive biases. Initially transformers have struggled to match CNN models for image classification [24], but are currently surpassing other models and are showing promising results in self-supervised scenarios and, more so than other types of architectures, transformers have shown impressive learning capacity and emergent behaviors under self-supervision [17].

Cosma and Radoi [12] were the first to propose GaitFormer, a direct adaptation of the vision transformer encoder model for gait recognition, utilizing individual skeletons as input “patches”, essentially only performing temporal attention, ignoring spatial attention relationships. GaitFormer was trained in a self-supervised fashion and surpassed other gait recognition methods even without any fine-tuning. Such previous work is encouraging and paves the way for a more in-depth study of the potential application of transformer architectures for gait analysis. Can vision transformer models be adapted for self-supervised learning of skeleton gait representations? The main architectural issue in vision transformers is defining the proper relationships between image patches, which define local and global information. When applied to gait, the choice of patch dimensions corresponds to the amount of encoded temporal and spatial information of the skeleton sequence.

In this work, we present an extensive study of five different vision transformers, adapted for gait recognition. We explore the classical ViT model [24], CaiT [25], CrossFormer [26], TwinsSVT [27], and token-to-token ViT [28]. Each architecture is trained separately in a contrastive self-supervised manner on two large-scale “in the wild” datasets of 2D gait skeleton sequences: DenseGait—an automatically gathered dataset from raw surveillance streams, and GREW, a smaller dataset but containing clean human annotations.

We explore transfer capabilities across two controlled datasets for gait recognition, CASIA [6], and FVG [29]. For each dataset, we analyze direct (zero-shot) transfer and data efficiency during fine-tuning by training with progressively larger subsets of the datasets. Moreover, we conduct an ablation study on the relationship between spatial and temporal dimensions for patch sizes for SimpleViT and CaiT, the standard backbones for most of the vision transformers to date.

The rest of the paper is organized as follows. We conduct a high-level overview of related works pertaining to gait recognition models and vision transformers. We observe that gait representation models highly benefit from self-supervised training to have more robust and general embeddings, and transformer models have shown great modeling capacity in self-supervised training regimes. Further, we mathematically describe the five architectures that we benchmark and describe the data preprocessing and skeleton transformations needed to be performed, such that vision transformers have to operate seamlessly on skeleton sequences. We also describe data augmentations, training and benchmarking datasets, and experimental setup. We showcase results on CASIA-B and FVG for each of the five architectures and the two ’pretraining in-the-wild’ datasets. Finally, we make an ablation study on the relationship between the spatial and temporal patch sizes and provide a brief discussion of our results. We make our source code publicly available on GitHub (https://github.com/cosmaadrian/gait-vit, accessed on 28 February 2023) for transparency and reproducibility.

## 2. Related Work

In this section, we make a brief overview of existing methods for gait recognition in controlled environments and "in the wild". Further, we describe main the developments of transformer models and, in particular, their application in the vision domain.

### 2.1. Gait Recognition

Similarly to face-based identification, gait recognition relies on metric learning. As opposed to traditional biometric authentication methods, which rely on a single image (e.g., face recognition) and require extensive cooperation (e.g., iris-based biometric authentication), gait features are processed as a sequence of motion snapshots. Such gesture dynamics require more complexity in determining the most informative sub-sequence but enable the use of unobtrusive authentication at a distance.

In this context, the task implies training an encoder network to map walking sequences to an embedding space where the embedding similarity corresponds to the similarity of the gait. Embeddings of walks that belong to the same person should be close to the embedding space and those who come from different identities need to be more distant. In this embedding space, inference can be made by obtaining the embedding of the gait sequence and utilizing the nearest-neighbor approach on a database of known walks.

Current approaches in gait-based recognition are divided into two categories: appearance-based [8,9] and model-based [10,12,30]. Appearance-based methods first obtain the silhouettes of the walking subjects with background subtraction or segmentation algorithms from each video frame. Then the sequence of silhouettes is fed into CNN-based architectures which extract spatial and temporal features which are aggregated into a final embedding for recognition. Model-based approaches extract the skeletons from RGB videos with pose estimation models [21,22]. Sequences of skeletons are usually processed by models which rely on graph convolutions [10,30] for obtaining the embedding of the gait.

GaitSet, the work of Chao et al. [8], regards the gait as an unordered set of silhouettes. The authors argue that this representation is more flexible than a silhouette sequence because it is robust to different arrangements of frames or the combination of multiple walking directions and variations. They utilize convolution layers for each silhouette in order to obtain image-level features and combine them into a set-level feature with Set Pooling. They obtain the final output by employing their own version of Horizontal Pyramid Matching [31].

Fan et al. [9] noticed the fact that specific parts of the human silhouette should have their own spatiotemporal expression as each one has a unique pattern. Their architecture, GaitPart, utilizes focal convolution layers (FConvs), which are a specialized type of convolution with a more restricted receptive field. The authors argue that the FConvs aid their architecture in learning more fine-grained features for different parts of the moving body. They also introduce the micro-motion capture modules, which are employed to extract the features of small temporal sequences.

Teepe et al. [30] propose GaitGraph, which leverages an adapted graph convolutional network called ResGCN [32] for encoding the spatiotemporal features obtained from the sequence of skeletons. Li et al. [33] propose PTP, which is a structure that aggregates multiple temporal features from one gait cycle based on their analysis of the most important stages of walking. They also utilize a graph convolutional network for spatial feature extraction, which works together with PTP. The authors introduce a novel data augmentation method that modifies the gait in order to have multiple paces in a cycle which is more realistic.

However, different from previous works, we are aiming to explore the performance of gait recognition architectures in self-supervised scenarios. Inspired by tremendous progress in the computer vision domain, we propose to adapt existing vision transformer architectures to operate on skeleton sequences instead of images and to test their modeling capacity in self-supervised scenarios. Most other works [8,9,30] focus their efforts on developing neural architectures that achieve impressive results on gait recognition on controlled datasets. However, we intend to remove the need for highly expensive manual annotations for gait datasets and explore ways in which self-supervised learning is appropriate for gait analysis pretraining. Previous works in this domain [10,12] showed potential for learning good gait representations from weakly annotated datasets. Cosma and Radoi [12] proposed GaitFormer, the first transformer-based architecture for processing skeleton sequences, inspired by the ViT [24] model. Similar to [12], we attempt to explore the performance of other vision transformer models, with different spatial and temporal dynamics in the patch processing mechanism. Large-scale datasets for gait recognition have been proposed in the past [7,12], which allows for the development of general architectures for representation learning.

### 2.2. Vision Transformers

While initially proposed for NLP tasks [16,34] with immense success, transformers have become widely utilized in computer vision in recent years [24,25,28,35,36,37]. Both domains have enjoyed unprecedented performances by using various variations of transformers, partially due to the increased model capacity and the transformers’ ability to benefit from self-supervision much more than previous models [17].

Dosovitskiy et al. [24] were the first to propose the utilization of transformer encoders for image classification, introducing the Vision Transformer (ViT). The architecture divides the input image into fixed-size patches of 16x16, flattens, and projects them with a linear layer to the embedding dimension. An extra class token (CLS) is inserted into the sequence and positional encodings are added to each vector. The resulting sequence of embeddings is given as input to a transformer encoder, which has the same structure as the one in [34] but uses the LayerNorm operator before each block instead of after (pre-norm). An MLP head is utilized in order to obtain the class label from the globally aggregated information in the class token.

The self-attention mechanism introduced by Vaswani et al. [34] takes a sequence of items as input and estimates the interaction between all of them by aggregating the global information for each individual element in the sequence. In order to compute different interactions between the elements of a sequence, the multi-head self-attention (MSA) module concatenates the results of multiple self-attention blocks and projects the output onto a learnable weight matrix. The transformer encoder introduced in [34] is composed of multiple stacked layers consisting of a MSA block, a feedforward (FFN) block, residual connections between each block, and a LayerNorm (LN) after each block.

Touvron et al. [25] propose two architectural changes in order to improve the performance of deep vision transformers. Their first contribution, LayerScale, facilitates the training of deeper models by adding a learnable diagonal matrix which is multiplied by the output of the residual blocks. Because the matrix is initialized with small values, it forces the results of the transformer encoder layers to have a small contribution to the output of the residual block at the beginning of training. Their second contribution is the class-attention mechanism. Instead of initially appending the CLS token, such as in the standard ViT, it is appended after a number of encoder blocks. After this stage, only the class token is updated and the patch tokens are kept frozen. This mechanism helps in decoupling the self-attention operations between patches from aggregating the information that will be used for classification.

Yuan et al. [28] argue that the simple tokenization of patches in the vanilla ViT has the limitation of not being able to model the local structure of the image and the interaction between neighboring patches. Consequently, they introduce a progressive tokenization process that combines neighboring tokens into a single one. This process consists of the Reshape module, which takes the sequence of tokens from the previous layer and constructs an image from them based on spatial closeness. The Soft Split module divides the constructed image into overlapping patches of tokens and feeds them to the next encoder. The generated tokens after the tokenization process are fed into a deep narrow ViT backbone for classification.

As noted by Wang et al. [35] the standard Vision Transformer was specifically designed for image classification and is not suited for other tasks such as object detection or segmentation. Because of this, they propose the Pyramid Vision Transformer (PVT) which takes inspiration from CNN architectures by producing intermediary feature maps with decreasing spatial dimensions and an increasing number of channels. This pyramid structure helps the model in learning multi-scale features which can be used for various tasks. The model first processes tokens were obtained from patches of dimensions 4 × 4, and at each stage, the tokens correspond to larger spatial dimensions of patches.

The computational cost of classic self-attention is $O(N^{2}\cdot d)$ where *N* is the number of tokens in the sequence and *d* is the vector dimension. The quadratic computational cost in terms of the number of tokens becomes a practical problem with increasing input image resolution since each token in a sequence corresponds to a patch in the image. In the literature, there are a number of techniques with which to reduce the computational cost of vanilla self-attention [26,35,36]. PVT [35] uses the spatial reduction attention, which reduces the spatial size of the Key and Value vectors before the self-attention with a reshaping operation and a linear projection.

The Swin transformer [36] which also has a pyramid structure replaces the self-attention block with a module that approximates it. The module groups neighboring patches in local windows and performs the self-attention operation only inside these windows. In order to communicate the information with other windows, it shifts the local windows so that they also contain patches from neighboring windows and computes self-attention again. Chu et al. [27] adopted the PVT architecture and proposed a similar method for approximating self-attention. They also performed local attention between patches in a window, similar to the Swin transformer. In order to communicate information with other windows, they conducted self-attention between a representative of each window and all other windows. CrossFormer [26] also builds upon the PVT. It utilizes short distance attention, which is similar to the local attention in the Swin transformer, but for leaking information to other windows it employs long-distance attention, which computes the interaction between patches, which have a fixed distance between them. It also combines multi-scale patches centred around the same pixel in order to obtain the tokens for the transformer blocks, which helps the model in learning cross-scale interactions.

Yang et al. [37] propose the focal attention mechanism for learning both short and long-range interactions between tokens which makes vision transformers able to process high-resolution images. For each image patch, the focal self-attention module computes interactions with spatially close patches and with summarized windows of patches that are more distant. The summarization of windows of patches is done via pooling and it captures less information when the patches are far away. The RegionViT [38] utilizes the PVT architecture and adds two tokenization paths for each feature map. The first tokenization path obtains regional tokens which consist of patches that cover a large number of pixels. The second tokenization path obtains local tokens which capture low-level information by containing few pixels. These two types of tokens are fed as input to the regional-to-local transformer encoder in which first self-attention between regions is computed, then between each regional token and its corresponding local tokens.

The LeViT architecture [39] combines both CNNs and the self-attention mechanism. An image is first fed into a CNN encoder, which decreases the spatial dimensions and increases the channel dimension. The resulting feature maps are fed into a hierarchical ViT that contains a shrinking attention module between its encoders in order to further decrease the spatial dimensions and increase the channel dimension of the feature maps. Architectures based on attention have also been employed in video-based tasks where temporal information needs to be taken into account. Architectures, such as ViViT [40] and TimeSformer [41], utilize the self-attention mechanism over both the spatial and the temporal dimensions. Because of this, the model learns to capture the spatial information from each individual frame and the change over time.

## 3. Method

In this section, we provide a detailed description of each architecture and chosen hyperparameters. Further, we describe the data processing and the proposed design decisions to adapt vision transformers to work with skeleton sequences. Lastly, we describe the initialization methods, evaluation protocol, and evaluation datasets.

### 3.1. Architectures Description

**Figure 1:**
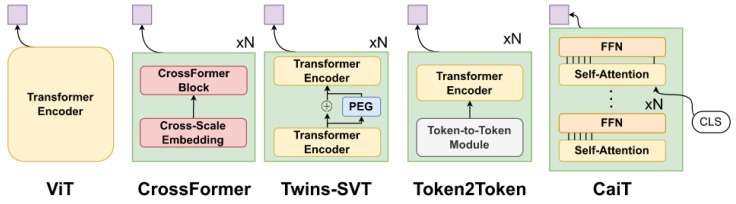
High-level overview of each of the five architectures in our study. The main particularities/blocks of each model is shown. The final embedding for the skeleton sequence is shown in mauve, which corresponds to average pooling across the sequence.

We explored five different variants of the Vision Transformers (Figure 1), which were developed for more optimized computation on images, in terms of downstream performance and inference time. In particular, we explore the classic ViT [24], CaiT [25], Token2Token ViT [28], Twins-SVT [27]. In general, the flavors of vision transformers are dealing with improvements upon the “classic” way of processing images with transformers, as proposed in ViT: images are split into equal-sized and non-overlapping patches which are flattened and projected into a lower dimensional space to be then treated as “tokens”, in a similar manner to NLP applications. In the case of gait analysis, a square patch corresponds to a group of joints that vary across a small temporal window.

The standard transformer encoder takes as input a sequence of items ($X\in R^{n\times d}$ where *n*—number of items, *d*—embedding dimension) and projects them onto three different learnable weight matrices obtaining the Queries ($Q\in R^{n\times d_{q}}$), the Keys ($K\in R^{n\times d_{k}},d_{k}=d_{q}$), and values ($V\in R^{n\times d_{v}}$), where $d_{q}$, $d_{k}$, and $d_{v}$ are the dimensions for the queries, keys and values, respectively. Attention is computed as:

(1) $$Attention\left(Q,K,V\right)=Softmax\left(QK^{T}/\sqrt{d_{k}}\right)V$$

Multi-head self-attention (MSA) employs multiple attention modules in order to compute different interactions between the elements of a sequence. Usually, positional encodings are added to the input to encode the order of the input tokens. An encoder layer is made up of a MSA block and a feedforward (FFN) block. Residual connections and LayerNorm (LN) are utilized after each block. Given a sequence of tokens ($z_{l}\in R^{n\times d}$) to the input of a transformer encoder layer, the output $z_{l+1}$ is computed as:

(2) $$\begin{array}{c} z_{l}^{\prime }=LN\left(z_{l}+MSA\left(z_{l}\right)\right)\\ z_{l+1}=LN\left(z_{l}^{\prime }+FFN\left(z_{l}^{\prime }\right)\right) \end{array}$$

For most architectures, we fixed the number of layers, attention heads, and feature dimensionalities whenever possible. As such, we choose 4 layers with 4 attention heads each, a dimension of 512 for the feedforward network, and a final embedding size of 128.

**ViT** The Vision Transformer [24] obtains an input sequence of tokens by dividing the image into patches and linearly projecting them to the embedding dimension. The resulting sequence along with an extra class token (CLS) are given as input to a transformer encoder. Moreover, the ViT encoder uses pre-norm, as opposed to post-normalization. The output of a layer can be computed as:

(3) $$\begin{array}{c} z_{l}^{\prime }=z_{l}+MSA\left(LN\left(z_{l}\right)\right)\\ z_{l+1}=z_{l}^{\prime }+FFN\left(LN\left(z_{l}^{\prime }\right)\right) \end{array}$$

For ViT, we chose a configuration with a spatial patch size of 64 and a temporal patch size of 1, essentially each patch being represented as a single skeleton. We delve into more detail about different patch configurations in Section 4.3.

**CaiT** The CaiT [25] architecture incorporates a learnable diagonal matrix which is multiplied with the output of the residual block. This facilitates the training of deeper vision transformers by enforcing a small residual contribution at the start of training. The output of a CaiT layer can be calculated with the following formula:

(4) $$\begin{array}{c} z_{l}^{\prime }=z_{l}+diag\left(\lambda_{l,1},\ldots ,\lambda_{l,d}\right)\times MSA\left(LN\left(z_{l}\right)\right)\\ z_{l+1}=z_{l}^{\prime }+diag\left(\lambda_{l,1}^{\prime },\ldots ,\lambda_{l,d}^{\prime }\right)\times FFN\left(LN\left(z_{l}^{\prime }\right)\right), \end{array}$$

where $\lambda_{l,i}$ and $\lambda_{l,i}^{\prime }$ are learnable parameters. The model also decouples the computation of interactions between input tokens from the computation of the class embedding which aggregates all the global information. This is done with class attention which introduces the CLS token to the input sequence after the interactions have been obtained and freezes all the other tokens. For the CaiT encoder, we used the same configuration as in ViT, but for the CLS encoder, we used a depth of 2 layers.

**Token2Token ViT** The Token2Token architecture [28] contains a progressive tokenization process that models the local structure of an image by combining neighboring tokens. The tokenization process first constructs an image-like structure from an input sequence of tokens with the help of the Reshape module. Then the image is divided into overlapping patches of tokens via the Soft Split (SS) module. The resulting output from the tokenization module is computed as:

(5) $$\begin{array}{c} z_{l}^{\prime }=FFN\left(MSA\left(z_{l}\right)\right)\\ I_{l}=Reshape\left(z_{l}^{\prime }\right)\\ z_{l+1}=SS\left(I_{l}\right) \end{array}$$

For Token2Token, we used 2 layers with patch sizes of {2, 8} and {2, 4} for the first layer, and {4, 16} for the second layer.

**Twins-SVT** The Twins-SVT [27] architecture replaces classic self-attention block with a module called spatially separable self-attention (SSSA) that approximate the operation. SSSA consists of a locally-grouped self-attention (LSA) which computes the interaction only between tokens inside the same local window and the global sub-sampled attention (GSA) which aggregates global information by doing self-attention between all representatives of each local window computed by convolving the neighboring tokens. The operations of a Twins layer can be written as:

(6) $$\begin{array}{c} z_{l}^{\prime }=z_{l}+LSA\left(LN\left(z_{l}\right)\right)\\ z_{l+1}=z_{l}^{\prime }+FFN\left(LN\left(z_{l}^{\prime }\right)\right)\\ z_{l+1}^{\prime }=z_{l+1}+GSA\left(LN\left(z_{l+1}\right)\right)\\ z_{l+2}=z_{l+1}^{\prime }+FFN\left(LN\left(z_{l+1}^{\prime }\right)\right) \end{array}$$

For the Token2Token encoder, we used dimensions of {16, 32, 64, 128} for the layers, uniform patch dimensions of 2, local patch sizes of 4 and global window of 4 for every layer.

**CrossFormer** CrossFormer [26] utilizes a cross-scale embedding layer, which concatenates patches of different sizes centred around the same pixel and linearly projects the result to the embedding dimension in order to obtain tokens. This helps the architecture in learning both low-level and high-level features. Similar to the case of the Twins architecture, the CrossFormer approximates self-attention with two complementary modules: short distance attention (SDA) and long distance attention (LDA). The SDA module works the same as the local attention in Twins and the LDA module computes the interactions between tokens at a certain fixed distance. The operations of the CrossFormer layers can be written as:

(7) $$\begin{array}{c} z_{l}^{\prime }=z_{l}+SDA\left(LN\left(z_{l}\right)\right)\\ z_{l+1}=z_{l}^{\prime }+FFN\left(LN\left(z_{l}^{\prime }\right)\right)\\ z_{l+1}^{\prime }=z_{l+1}+LDA\left(LN\left(z_{l+1}\right)\right)\\ z_{l+2}=z_{l+1}^{\prime }+FFN\left(LN\left(z_{l+1}^{\prime }\right)\right) \end{array}$$

For the CrossFormer encoder, we used dimensions of {16, 32, 64, 128} for the layers, global window sizes of {4, 2, 2, 1}, local window size of 2, cross-embedding strides of 2, and cross-embedding kernel sizes of {{2, 4, 8, 16}, {2, 4}, {2, 4}, {2, 4}}.

### 3.2. Data Preprocessing

For both DenseGait and GREW datasets, we employ the same preprocessing procedure. For each extracted and tracked skeleton sequence containing 18 joints with *x*, *y* coordinates and an additional confidence score, we first normalize the sequence by centring at the pelvis coordinates ($x_{pelvis}$, $y_{pelvis}$) and by scaling horizontally and vertically, according to human body proportions (i.e., the distance between shoulders: $|x_{R.shoulder}-x_{L.shoulder}|$ and the distance from the neck to the pelvis: $|y_{neck}-y_{pelvis}|$). For each coordinate ($x_{joint}$, $y_{joint}$) of each of the 18 joints in the COCO pose format, we apply the following normalization procedure:

(8) $$x_{joint}=\frac{x_{joint}-x_{pelvis}}{|x_{R.shoulder}-x_{L.shoulder}|}$$

(9) $$y_{joint}=\frac{y_{joint}-y_{pelvis}}{|y_{neck}-y_{pelvis}|}$$

Through the normalization process, differences between camera resolutions and the subject’s distance from the camera are eliminated. Moreover, we eliminate appearance information regarding the height and width of a subject, which do not pertain to movement information. This step is similar to the alignment step in modern face recognition models [42]. Moreover, we also employ a batch normalization [43] layer at the beginning of each model to further normalize the resulting image.

**Figure 2:**
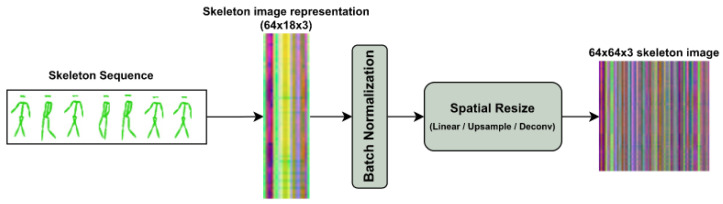
Preprocessing procedure. Skeleton sequences are treated as an image that is sent through a batch normalization layer. To transform the image square dimensions, we propose several methods for spatially resizing the skeleton sequence.

Given the temporal dimension *T* (i.e., number of frames) and the skeleton spatial dimension *J* (i.e., number of joints), naive skeleton sequences are encoded as images of shape (T,J,3), where, in our case, T=64 and J=18. Most vision transformers, however, assume that the images are square. Therefore, we propose multiple variants of resizing the spatial dimension such that the image is transformed to (T,T,3), which is equivalent to artificially increasing the number of joints (see Figure 2).

**Figure 3:**
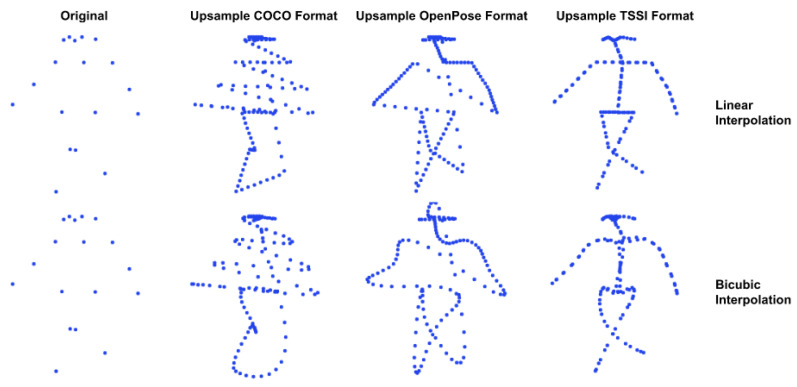
Example of upsampling the skeletons using traditional image resizing methods. A naive skeleton representation does not provide an interpretable result. By utilizing the tree structure skeleton image (TSSI) format, spatial resizing corresponds to artificially increasing the number of joints in a skeleton.

We use a simple upsample to interpolate between neighboring joints (Figure 3). However, naively doing so on the skeleton results in spurious joints across the skeleton, regardless of the choice of skeleton formats (e.g., OpenPose or COCO), since the joint ordering does not preserve any semantic meaning. This observation is in line with the work of Yang et al. [44], which proposes a tree structure skeleton image (TSSI) to propose the spatial relationships between joints. It is based on a depth-first tree traversal ordering of joints, which preserves the skeleton’s structural information. Figure 3 (right), showcases the effects of different skeleton formats and upsample methods. For this resizing method, we used TSSI format and bicubic interpolation.

Furthermore, we experimented with two upscaling methods, which were learnable during training. We used a simple linear layer applied to each flattened skeleton to increase the number of joints. This is the most straightforward manner to transform each skeleton, but it does not account for any spatial relationships between joints. To address this, we also employ a set of 2D deconvolutional layers on the skeleton sequence for resizing while also taking the structural information into account; for this method, we also employ the TSSI format.

Table 1 showcases the results for each resizing method for all architectures. The models were trained and evaluated on CASIA-B for 200 epochs, and we show results for normal walking. For the rest of our experiments, we chose to upsample the skeleton sequence with bicubic interpolation.

### 3.3. Training Details

While there are several possible self-supervised pretraining procedures, we opted for a contrastive pretraining approach since it is the same procedure for the actual retrieval task of gait recognition. Contrastive approaches encourage representations belonging to the same class to be close in latent space, while simultaneously being distant from representations belonging to different classes. In particular, we use Supervised Contrastive [45] for pretraining. SupCon loss operates on a multi-viewed batch: each sample in the batch has multiple augmented versions of itself. It was shown to naturally be more robust to data corruption, it alleviates the need for careful selection of triplets since the gradient encourages learning from hard examples and is less sensitive to hyperparameters.

Let i∈I≡{1⋯2N} be the index of an arbitrary augmented sample. SupCon loss is defined as

(10) $$L^{sup}=\sum_{i\in I}\frac{-1}{\left|P(i)\right|}\sum_{p\in P(i)}\log\frac{\exp(z_{i}\cdot z_{p}/\tau )}{\sum_{a\in A(i)}\exp\left(z_{i}\cdot z_{a}/\tau \right)}$$

Given a encoder backbone Enc(·), we extract $z_{l}=Proj\left(Enc\left(x_{l}\right)\right)$, the projected embedding for skeleton sequence $x_{l}$. The anchor indices are A(i)≡I\{i} and the positive indices are defined as $P\left(i\right)\equiv \{p\in A\left(i\right):{\tilde{y}}_{p}={\tilde{y}}_{i}\}$. We used a two-view sampler, in which each skeleton sequence is augmented in two different ways. One important parameter for SupCon loss is the temperature τ, which substantially affects optimization. Lower temperatures improve training performance, as it is equivalent to optimizing for hard examples [45], while higher values promote loss smoothness as samples are better separated. We chose a temperature of τ=0.001 in all our experiments. As indicated by Chen et al. [46], we utilize a linear projection layer to a lower dimensional space, to diminish the course of dimensionality. Particularly, we linearly project the gait embedding as outputted by the encoder Enc(·) into a 128-dimensional space with a simple feedforward layer Proj(Enc(·)).

Furthermore, in all our experiments we employ an Adam optimizer with a batch size of 256 and with a variable learning rate ranging from 0.001 to 0.0001 using a triangular cyclical learning rate schedule [47]. Experiments were performed on 2x NVIDIA RTX 3060 GPUs, with 12 GB of VRAM each.

### 3.4. Data Augmentation

Training in a self-supervised contrastive manner with SupCon loss implies the use of data augmentation to provide multiple augmented “views” of the same skeleton sequence. Augmentations used for our walking skeleton sequences are in line with other works in this area [10,12,30]. The main augmentation used is random temporal cropping with a period length of T=64 frames. Given that skeletons are tracked for a variable duration of time, we use cropping to ensure that all sequences have the same length. Moreover, a walking person might change direction and perform other actions across the tracked duration; consequently, cropping induces more variability for the same sequence.

Furthermore, we modify the walking pace by slowing down or speeding up the walk. We used speed modifiers of {0.5,0.75,1,1.25,1.5,1.75,2.0}. This is adapted from the work of Wang et al. [48] for self-supervised learning of video representations. Moreover, pace modification has been used in gait analysis in the past [33].

We also use random flipping with a probability of 0.5, sequence reversal with a probability of 0.5, additive Gaussian noise for each joint with σ=0.005, and random dropout of 5% of joints with a probability of 0.01 to simulate missing joints from the pose estimation model.

### 3.5. Initialization Methods

To gauge the impact of self-supervised pretraining performance on the proposed architectures, we explore three different initialization methods. Table 2 showcases the various datasets used in the literature. While CASIA-B [6] and FVG [29] are controlled datasets, mostly used for evaluation, we use DenseGait [12] and GREW [7] for self-supervised pretraining for the five architectures. DenseGait and GREW are two of the largest realistic gait databases, collected in outdoor settings, which presumably contain the majority of gait variations, behaviors, and complexities present in everyday life. We choose these datasets to have more general gait representations, to allow for gait authentication in general surveillance scenarios.

**DenseGait Pretraining** DenseGait is a large-scale “in the wild” gait dataset collected automatically from surveillance streams. It contains 217 K tracked skeleton sequences, extracted using AlphaPose [22], in various environments and camera angles from multiple geographical locations. Since DenseGait is collected automatically, its annotations in terms of the tracking identifier are noisy, and the dataset might contain sequences pertaining to the same subject, although this is a rare occurrence. However, this is the case for the majority of large-scale unlabeled datasets that contain samples belonging to the same semantic class, which is considered unrelated during training. We pretrain each architecture on DenseGait and use the trained parameters for further downstream performance evaluation of the controlled datasets.

**GREW Pretraining** GREW is another outdoor “in the wild” dataset but is carefully annotated such that it contains walking subjects across multiple days and different types of clothing. However, to conform to the requirements of the self-supervised regime, we discard the annotations and treat each walking sequence as a separate person. GREW is 2× smaller than DenseGait, containing 128 K skeleton sequences, while also having each pedestrian tracked for a smaller average duration [12]. We also pretrain each architecture on GREW and use the trained parameters for downstream performance evaluation.

**Random Initialization** This initialization method corresponds to no pretraining (i.e., training from scratch). Each architecture is trained with random weight initialization on the downstream datasets. This method is a baseline to compare the performance gains by pretraining.

### 3.6. Evaluation

Downstream task performance is evaluated in two different manners for gait recognition. We directly test the retrieval capabilities of pretrained architectures, without fine-tuning for a specific task. This method corresponds to zero-shot transfer. Further, we fine-tune each architecture using a 10× smaller learning rate than during pretraining, with a progressively smaller learning rate at the beginning of the network, corresponding to layer-wise learning rate decay (LLRD) [50] policy.

The evaluation of the downstream performance is performed on two popular gait recognition datasets: CASIA-B [6] and FVG [29]. Both datasets feature a small number of subjects under strict walking protocols, which are controlled over various confounding factors: camera angle, clothing, accessories, and walking speed.

**CASIA-B** is an indoor dataset containing 124 subjects captured from 11 synchronized cameras. Each individual walks in three different conditions: normal walking, clothing change, and bag carrying. Since its release, it has been a staple for benchmarking gait analysis models, being one of the most used datasets in this area. We use the first 62 subjects as the training set and the remainder of the 62 for performance evaluation. For gait recognition, we choose to evaluate performance on a per-angle basis in a “leave-one-out” setting, in which the gallery set contains all walking angles except the probe angle.

**Front-View Gait** is another popular dataset for gait recognition, having 226 subjects walking outdoors under various protocols. Different from CASIA-B, FVG features additional confounding factors: walking speed, cluttered background, and the passage of time (i.e., some subjects have registered walks that span a year). Furthermore, all subjects are captured with a front-facing camera angle, which is considered the most difficult angle for gait recognition since it contains the smallest amount of perceived joint movement variation. We used the first 136 subjects for training and the rest for performance evaluation. Performance evaluation for gait recognition adheres to the protocols outlined by the authors, in which we use the normal walking sequence in the gallery set and use the other conditions in the probe set.

For all evaluation scenarios, we use deterministic cropping in the centre of the walking sequence and do not use any test-time augmentations.

## 4. Experiments and Results

### 4.1. Evaluation of CASIA-B

**Figure 4:**
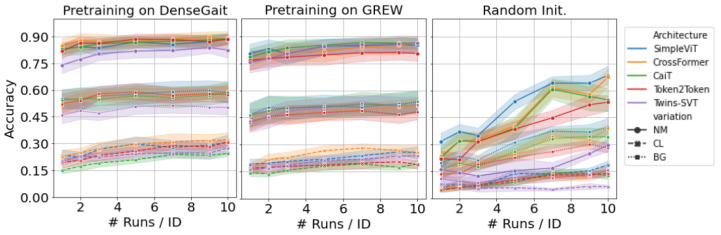
Results on CASIA-B after fine-tuning each architecture on progressively larger samples of the training data. Models are pretrained in a self-supervised manner on DenseGait (left-hand side) and GREW (centre). Lines represent average accuracy over all angles, shades represent standard deviation values. Compared to random initialization (right-hand side), pretraining on diverse datasets offers a significant boost of performance, especially in low-data regimes. Best viewed in color.

We pretrain each architecture on DenseGait and GREW, respectively, and evaluate the performance on CASIA-B and FVG. In the first set of experiments, we are interested in evaluating the performance on CASIA-B in a fine-tuning scenario after pretraining, with progressively larger training samples. We train each network on the first 62 identities, with all available walking variations, and kept the rest for testing. Recognition evaluation was performed using the first 4 normal walking samples as the gallery set, and the rest as a probe set. Figure 4 showcases the accuracy for each of the three walking variations (normal—NM, clothing—CLm and carry bag—BG) for CASIA-B. For this scenario, we randomly sampled K={1,2,3,5,7,10} walks per subject, per angle, and trained the model. While the performance is relatively similar between architectures, it is clear that pretraining offers a significant boost in performance compared to random initialization, regardless of the pretraining dataset choice. Moreover, SimpleViT, CrossFormer, and Twins-SVT seem to have similar high performance, while Token2Token is slightly lagging. This suggests that the progressive tokenization method used in Token2Token, which was specifically designed for image-like structures, does not effectively capture the characteristics of gait sequences. There is a noticeable difference between the pretraining datasets: DenseGait seems to offer a consistent performance increase in the two walking variations (CL and BG) when compared to GREW. This is indicative of the fact that DenseGait includes more challenging and realistic scenarios that better prepare the model for conditions where the walking pattern is affected by external factors.

Table 3 showcases a fine-grained comparison between all the tested architectures on CASIA-B. We compared the performance for pretrained networks in a zero-shot (i.e., direct transfer) scenario and a fine-tuned scenario with 100% of the available training data. We also included a randomly initialized network trained from scratch on CASIA-B. Consistent with results from Figure 4, we find that the difference between the pretraining dataset in the zero-shot scenario is largely reduced when fine-tuned with all the available data, which is an indication of the fact that the fine-tuning process is able to adapt the weights of the models to the distribution of the dataset. Furthermore, SimpleViT, CrossFormer, and Twins-SVT consistently outperform CaiT and Token2Token across variations.

**Figure 5:**
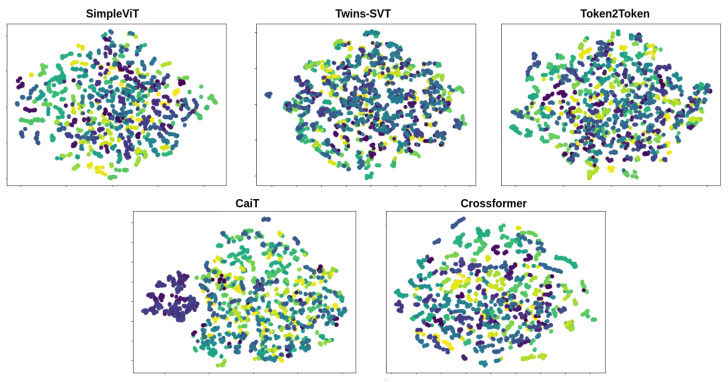
Qualitative visualizations of the network embeddings after fine-tuning on CASIA-B for each architecture using t-SNE projections. Each color represents an individual identity. Multiple dots per identity represent the various walking viewpoints. We omit uninformative axis dimensions.

The architectures are trained to map walking sequences to an embedding space, where the proximity between points reflects the similarity of the corresponding walking sequences. This means that the embeddings of unseen gait sequences from the same identity should be close to each other in the embedding space and form clusters, while the embeddings of different identities should be further away from each other and form distinct clusters. This is important as it allows the model to generalize to unseen gait sequences and accurately identify the individual by employing a nearest-neighbor approach. In Figure 5, we present the clustering for the embeddings for each identity in the test set of CASIA-B after dimensionality reduction with t-SNE [51]. We used the 256-dimensional embedding vector and project it into two dimensions. SimpleViT and CrossFormer seem to have the best separation of identities, irrespective of camera viewpoint.

### 4.2. Evaluation of FVG

**Figure 6:**
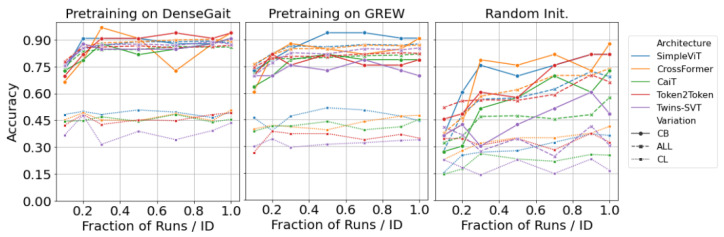
Results on FVG after fine-tuning each architecture on progressively larger samples of the training data. Models are pretrained in a self-supervised manner on DenseGait (left-hand side) and GREW (centre). Compared to random initialization (right-hand side), pretraining on diverse datasets offers a significant boost of performance, especially in low-data regimes. Best viewed in color.

Similarly, we evaluated the performance of each architecture on FVG, which is qualitatively different from CASIA-B, as it contains only a single viewing angle. We fine-tuned each pretrained network on a fraction f={0.1,0.2,0.3,0.5,0.7,1.0} of the 12 runs per person in the training set. Fine-tuning results are presented in Figure 6. Results follow a similar trend to those for CASIA-B: SimpleViT and CrossFormer have consistently high performances, and the use of a pretraining dataset is clearly beneficial for downstream performance. Further, pretraining on DenseGait seems to carry on a constant accuracy improvement. As noted by Cosma and Radoi [12], DenseGait contains subjects tracked for a longer duration, and this provides more variation in the contrastive learning objective, similar to random cropping for self-supervised pretraining for natural images. Similar to the results on CASIA-B, the clothing variation severely lags behind the normal walking scenario.

In Table 4, we present more fine-grained results on the testing set of FVG between pretrained models, similar to the CASIA-B scenario. Pretraining results are consistent: pretraining on DenseGait directly correlates to the improvement in the downstream accuracy. While pretraining on both datasets improves performance in all scenarios, the improvement is particularly significant in the CBG (Cluttered Background) scenario which usually consists of having more people in the video, similar to what would be expected in realistic settings. This improvement likely comes from the fact that DenseGait and GREW were gathered in natural, uncontrolled environments, making them more realistic and challenging, thus better preparing the model for similar conditions to the ones in the CBG scenario. The ranking between models is similar to CASIA-B: SimpleViT, CrossFormer, and Twins-SVT consistently outperform CaiT and Token2Token. For both CASIA-B and FVG, CaiT slightly lags behind other models.

### 4.3. Spatiotemporal Sensitivity Test

**Figure 7:**
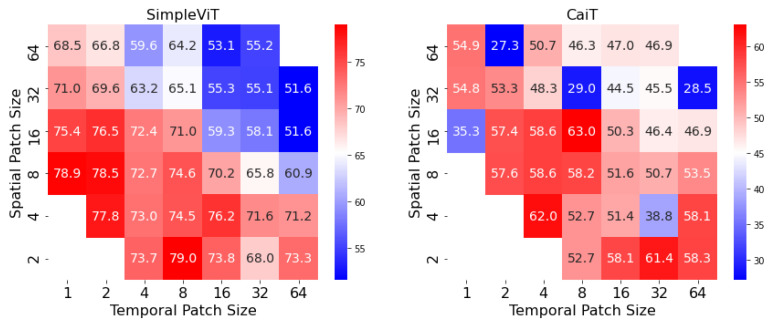
Sensitivity test on SimpleViT and CaiT for different configurations of patch sizes. Spatial patch size corresponds to the number of skeletal joints included in a particular patch, while temporal patch size corresponds to the number of frames in a patch. Since smaller patch sizes require more GPU memory to process, missing cells represent out-of-memory errors.

One particularity of vision transformers is the arbitrary choice of patch dimensions, which can prove to be crucial in the final performance. In the case of image processing, patch dimensions are not especially important, due to the translational invariance of the semantic content in an image. For skeletons sequences, however, patch dimensions correspond to specific and interpretable features of the input: patch height represents the amount of spatial information contained in a patch (i.e., number of skeletal joints included), while the temporal dimensions represent the amount of included temporal information (i.e., number of frames). The balance between the two should be carefully considered in the use of adapted vision transformers for gait analysis. In Figure 7, we showcase a heatmap in which every cell is the performance of a trained model (randomly initialized) on CASIA-B for normal walking. We train each model for 50 epochs for a fair comparison, and to gauge the convergence speed at a fixed number of steps. We constructed two heatmaps, one for SimpleViT and one for CaiT, since they have a similar underlying backbone, and it is straightforward to modify the patch sizes. The same process can be performed for the other tested architectures. We conclude that smaller patch sizes correspond to a positive increase in modeling performance for skeleton sequences, while the trade-off between spatial and temporal dimensions is not crucial, since performance is similar—the heatmap matrix is fairly symmetric by the second diagonal. Therefore, smaller square patch sizes such as (2,4) across spatial and temporal dimensions fare best for this task, while larger patch sizes such as (32,32) contain too little discriminative information. However, smaller patch sizes imply increasing the number of patches, which does require more computing power. For our setup of two NVIDIA RTX 3060 GPUs, we reported out-of-memory errors for some combinations of smaller patch sizes.

The most likely reason for the improved performance with smaller patch sizes is that the architecture can better capture the complexity of the walking pattern by computing more intricate interactions between patches. Patches with the largest possible spatial sizes and smallest possible temporal sizes can be considered full representations of skeletons, whereas patches with the largest possible temporal sizes and smallest possible spatial sizes capture the entirety of an individual joint’s movements. As can be observed, the highest accuracy is achieved when the patch size incorporates a balance of both spatial and temporal information, which correspond to small movements of closely connected joints.

## 5. Discussion and Conclusions

In this work, we provided a comprehensive evaluation of five popular variants of the vision transformer adapted for skeleton sequence processing. Our efforts are in line with the recent advancements in deep learning to essentially unify the different modalities under the transformer architecture. We proposed a spatial upsampling method for skeletons (bicubic upsampling with TSSI skeleton format) to artificially increase the number of joints, such that the sequence can be properly consumed by the transformer encoders. Furthermore, each architecture was trained under the self-supervision training paradigm on two general and large-scale gait datasets (i.e., DenseGait and GREW), and subsequently evaluated on two datasets for gait recognition in controlled environments (i.e., CASIA-B and FVG). We chose to adopt the self-supervised learning paradigm to obtain general gait features, not constrained to a particular walking variation or camera viewpoint.

Our results imply the need for high quantity, high quality, and diverse datasets for pretraining gait analysis models. We showed that pretraining on DenseGait offers consistent accuracy improvements over GREW, due to the increase in size, the number of variations, and the average walking duration [12]. The most significant benefit, however, is in situations with low amounts of training data available. Our results show that training from scratch leads to significantly worse results than fine-tuning even with modest amounts of data (i.e., 10 sequences per person). Currently, most gait approaches are performed indoors in strictly controlled environments, which cannot generalize to the complexities of real-world interactions. Diverse training datasets are crucial for performing accurate in-the-wild behavioral analysis, especially since gait is a biometric feature easily influenced by external environmental factors, as well as internal and emotional components.

Our ablation study shows that smaller spatial-temporal patches are beneficial for better downstream results. This insight informs future developments of architectures for skeleton sequences, which have previously relied on processing an individual skeleton on a single patch [12].

Alongside concurrent efforts to bring gait analysis into realistic settings, our work further enables the transition of gait authentication and behavioral analysis from indoor, controlled environments to outdoor, real-world settings. In-the-wild gait recognition will become ubiquitous with the developments of smart sensors and efficient neural architectures that process motion-driven behavior in real time.

## Figures and Tables

**Table 1:** Results for normal walking (NM) by training on CASIA-B utilizing different resizing methods for the skeleton sequence.

	Architecture				
**Resize Type**	**ViT**	**CrossFormer**	**TwinsSVT**	**Token2Token**	**CaiT**
**Linear**	75.80	68.40	66.86	77.71	49.63
**Deconv**	73.90	65.24	**70.96**	71.26	55.05
**Upsample Bilinear**	80.49	78.66	67.66	**79.32**	**55.20**
**Upsample Bicubic**	**80.57**	**81.01**	62.60	78.73	50.36

**Table 2:** Comparison of existing datasets for gait analysis. FVG, CASIA-B, and OU-ISIR are collected in controlled environments and are mainly used for benchmarking. GREW, UWG, and DenseGait are collected in realistic environments and offer more walk variability. * Number of IDs is approximate, as given by the pose tracker.

Dataset	# IDs	Sequences	Views	Env.	Note
FVG [29]	226	2857	1	Outdoor	Controlled
CASIA-B [6]	124	13,640	11	Indoor	Controlled
OU-ISIR [49]	10,307	144,298	14	Indoor	Controlled
GREW [7]	26,000	128,000	-	Outdoor	Realistic
UWG [10]	38,502 *	38,502	-	Outdoor	Realistic
DenseGait [12]	217,954 *	217,954	-	Outdoor	Realistic

**Table 3:** Fine-grained comparison for each architecture on CASIA-B, for each of the three walking variations and 11 viewpoints. For each model, we compared the performance between zero-shot (*ZS*) and Fine-Tuned (*FT*) for models pretrained on DenseGait and GREW, respectively, and no pretraining (*Rand. Init.*).

	Method	Kind	Pretraining Dataset	0${}^{\circ }$	18${}^{\circ }$	36${}^{\circ }$	54${}^{\circ }$	72${}^{\circ }$	90${}^{\circ }$	108${}^{\circ }$	126${}^{\circ }$	144${}^{\circ }$	162${}^{\circ }$	180${}^{\circ }$	Mean
**NM**	SimpleViT	Rand. Init.	−	73.39	82.26	81.45	76.61	55.65	51.61	62.10	70.97	72.58	66.94	59.68	68.48
	SimpleViT	ZS	DenseGait	69.35	62.90	69.35	71.77	48.39	49.19	50.00	68.55	71.77	72.58	58.06	62.90
	SimpleViT	ZS	GREW	59.68	76.61	70.16	69.35	38.71	40.32	47.58	64.52	63.71	60.48	54.03	58.65
	SimpleViT	FT	DenseGait	82.26	91.94	97.58	95.16	82.26	86.29	89.52	90.32	88.71	91.13	82.26	88.86
	SimpleViT	FT	GREW	88.71	91.13	91.94	92.74	79.03	72.58	82.26	83.87	87.10	92.74	74.19	85.12
	CaiT	Rand. Init.	−	65.32	70.97	58.87	51.61	39.52	49.19	58.06	68.55	55.65	42.74	43.55	54.91
	CaiT	ZS	DenseGait	76.61	67.74	70.16	60.48	48.39	61.29	65.32	64.52	75.00	75.00	61.29	65.98
	CaiT	ZS	GREW	70.97	66.13	67.74	62.10	39.52	37.90	37.10	50.81	54.03	65.32	54.84	55.13
	CaiT	FT	DenseGait	87.90	91.94	97.58	91.94	87.10	82.26	90.32	93.55	90.32	87.10	78.23	88.93
	CaiT	FT	GREW	85.48	91.94	92.74	93.55	76.61	81.45	88.71	85.48	82.26	86.29	84.68	86.29
	Token2Token	Rand. Init.	−	50.00	58.87	58.87	58.06	51.61	53.23	58.06	54.03	61.29	54.03	31.45	53.59
	Token2Token	ZS	DenseGait	61.29	79.84	73.39	74.19	54.84	43.55	54.84	74.19	71.77	83.87	79.84	68.33
	Token2Token	ZS	GREW	54.03	69.35	75.00	66.94	26.61	22.58	33.06	60.48	57.26	48.39	49.19	51.17
	Token2Token	FT	DenseGait	80.65	90.32	93.55	93.55	90.32	85.48	91.13	92.74	89.52	90.32	76.61	88.56
	Token2Token	FT	GREW	70.16	83.87	91.13	90.32	78.23	76.61	82.26	83.87	80.65	80.65	67.74	80.50
	Twins-SVT	Rand. Init.	−	33.87	41.94	33.06	34.68	21.77	30.65	26.61	29.84	30.65	21.77	20.97	29.62
	Twins-SVT	ZS	DenseGait	66.94	56.45	71.77	64.52	45.16	42.74	50.81	70.16	72.58	66.94	68.55	61.51
	Twins-SVT	ZS	GREW	50.81	70.97	66.13	60.48	36.29	33.87	29.03	54.03	59.68	51.61	47.58	50.95
	Twins-SVT	FT	DenseGait	71.77	85.48	87.10	87.90	74.19	83.87	79.03	89.52	88.71	88.71	70.97	82.48
	Twins-SVT	FT	GREW	74.19	88.71	95.16	91.94	86.29	82.26	88.71	87.10	90.32	92.74	72.58	86.36
	CrossFormer	Rand. Init.	−	65.32	67.74	78.23	78.23	65.32	70.97	71.77	70.16	68.55	56.45	49.19	67.45
	CrossFormer	ZS	DenseGait	87.10	91.94	95.97	91.94	70.16	60.48	72.58	86.29	89.52	83.87	75.81	82.33
	CrossFormer	ZS	GREW	54.84	63.71	62.90	61.29	54.03	40.32	25.00	41.94	37.90	42.74	39.52	47.65
	CrossFormer	FT	DenseGait	75.00	94.35	95.97	93.55	85.48	87.90	91.13	92.74	95.97	92.74	68.55	88.49
	CrossFormer	FT	GREW	75.81	88.71	95.16	91.13	92.74	85.48	88.71	88.71	89.52	86.29	69.35	86.51
**CL**	SimpleViT	Rand. Init.	−	20.97	23.39	19.35	15.32	13.71	12.90	16.94	18.55	19.35	27.42	16.13	18.55
	SimpleViT	ZS	DenseGait	12.90	29.03	23.39	16.13	10.48	11.29	13.71	17.74	22.58	15.32	18.55	17.37
	SimpleViT	ZS	GREW	7.26	20.97	12.10	8.06	8.06	6.45	11.29	18.55	13.71	12.90	6.45	11.44
	SimpleViT	FT	DenseGait	31.45	43.55	40.32	33.06	27.42	20.16	27.42	30.65	29.84	22.58	32.26	30.79
	SimpleViT	FT	GREW	34.68	33.87	26.61	20.16	16.94	17.74	25.81	27.42	20.97	31.45	25.00	25.51
	CaiT	Rand. Init.	−	12.90	20.16	11.29	10.48	10.48	7.26	12.10	16.13	9.68	17.74	12.10	12.76
	CaiT	ZS	DenseGait	6.45	19.35	11.29	11.29	4.84	11.29	20.16	12.10	15.32	12.10	10.48	12.24
	CaiT	ZS	GREW	8.06	20.97	8.87	13.71	6.45	9.68	10.48	14.52	9.68	12.10	7.26	11.07
	CaiT	FT	DenseGait	25.00	30.65	29.84	21.77	20.16	20.97	24.19	24.19	22.58	25.00	25.00	24.49
	CaiT	FT	GREW	18.55	16.94	21.77	16.94	13.71	18.55	25.00	20.16	19.35	26.61	14.52	19.28
	Token2Token	Rand. Init.	−	8.87	17.74	17.74	12.90	9.68	15.32	16.13	19.35	16.13	11.29	8.87	14.00
	Token2Token	ZS	DenseGait	8.87	25.81	15.32	16.13	4.03	11.29	14.52	18.55	20.16	15.32	16.13	15.10
	Token2Token	ZS	GREW	9.68	20.16	20.97	19.35	7.26	4.84	8.87	9.68	8.87	8.06	8.06	11.44
	Token2Token	FT	DenseGait	21.77	33.06	31.45	29.03	29.03	36.29	30.65	31.45	30.65	35.48	31.45	30.94
	Token2Token	FT	GREW	16.13	16.13	22.58	20.16	18.55	20.97	24.19	21.77	17.74	19.35	10.48	18.91
	Twins-SVT	Rand. Init.	−	7.26	4.03	6.45	7.26	4.84	8.06	9.68	6.45	6.45	6.45	5.65	6.60
	Twins-SVT	ZS	DenseGait	8.87	25.81	15.32	15.32	11.29	8.87	13.71	15.32	15.32	11.29	14.52	14.15
	Twins-SVT	ZS	GREW	9.68	24.19	8.06	10.48	12.10	8.87	8.87	16.13	11.29	11.29	7.26	11.66
	Twins-SVT	FT	DenseGait	23.39	33.06	33.87	27.42	21.77	25.00	29.03	34.68	29.03	27.42	17.74	27.49
	Twins-SVT	FT	GREW	20.16	26.61	24.19	29.84	21.77	21.77	29.03	29.84	20.16	22.58	16.94	23.90
	CrossFormer	Rand. Init.	−	9.68	17.74	18.55	18.55	18.55	18.55	17.74	21.77	12.10	11.29	12.10	16.06
	CrossFormer	ZS	DenseGait	16.94	29.84	23.39	20.16	11.29	8.06	16.94	20.97	15.32	19.35	18.55	18.26
	CrossFormer	ZS	GREW	16.94	14.52	13.71	11.29	7.26	8.87	11.29	11.29	11.29	11.29	8.87	11.51
	CrossFormer	FT	DenseGait	25.00	42.74	39.52	40.32	29.84	25.00	32.26	37.90	33.06	26.61	20.97	32.11
	CrossFormer	FT	GREW	20.97	29.03	39.52	23.39	23.39	22.58	25.81	31.45	24.19	26.61	15.32	25.66
**BG**	SimpleViT	Rand. Init.	−	46.77	55.65	50.00	44.35	31.45	40.32	30.65	35.48	37.90	36.29	26.61	39.59
	SimpleViT	ZS	DenseGait	54.03	53.23	55.65	45.16	37.90	25.81	37.10	45.16	44.35	44.35	40.32	43.91
	SimpleViT	ZS	GREW	49.19	50.81	50.81	46.77	41.13	25.81	27.42	43.55	36.29	29.84	29.03	39.15
	SimpleViT	FT	DenseGait	58.87	73.39	78.23	69.35	51.61	54.84	50.81	53.23	55.65	53.23	44.35	58.51
	SimpleViT	FT	GREW	61.29	68.55	70.97	61.29	47.58	44.35	49.19	42.74	49.19	53.23	38.71	53.37
	CaiT	Rand. Init.	−	46.77	43.55	45.97	34.68	20.97	28.23	33.06	32.26	35.48	29.03	25.00	34.09
	CaiT	ZS	DenseGait	60.48	55.65	58.06	41.13	35.48	28.23	37.10	47.58	49.19	50.81	42.74	46.04
	CaiT	ZS	GREW	55.65	45.16	55.65	37.90	35.48	20.97	20.16	31.45	40.32	29.03	32.26	36.73
	CaiT	FT	DenseGait	74.19	71.77	75.81	58.87	49.19	54.03	52.42	55.65	54.03	55.65	45.97	58.87
	CaiT	FT	GREW	62.10	66.94	73.39	58.87	53.23	48.39	51.61	42.74	49.19	50.81	38.71	54.18
	Token2Token	Rand. Init.	−	28.23	33.06	37.90	30.65	32.26	22.58	29.03	24.19	28.23	24.19	20.16	28.23
	Token2Token	ZS	DenseGait	45.97	61.29	66.13	52.42	35.48	25.00	35.48	47.58	53.23	55.65	53.23	48.31
	Token2Token	ZS	GREW	42.74	46.77	49.19	41.94	12.10	15.32	17.74	34.68	37.90	29.84	27.42	32.33
	Token2Token	FT	DenseGait	66.13	70.16	70.16	61.29	57.26	52.42	55.65	56.45	46.77	51.61	44.35	57.48
	Token2Token	FT	GREW	50.81	55.65	59.68	50.81	41.13	37.90	47.58	50.00	48.39	53.23	34.68	48.17
	Twins-SVT	Rand. Init.	−	20.16	26.61	24.19	18.55	12.90	12.10	10.48	12.10	9.68	12.90	12.90	15.69
	Twins-SVT	ZS	DenseGait	45.97	50.00	49.19	38.71	25.00	31.45	33.87	38.71	50.81	47.58	48.39	41.79
	Twins-SVT	ZS	GREW	37.90	45.16	39.52	30.65	25.81	13.71	18.55	24.19	28.23	33.87	27.42	29.55
	Twins-SVT	FT	DenseGait	45.97	56.45	61.29	50.00	43.55	37.10	53.23	52.42	50.81	58.87	42.74	50.22
	Twins-SVT	FT	GREW	59.68	58.06	64.52	54.84	46.77	49.19	50.81	52.42	43.55	56.45	37.90	52.20
	CrossFormer	Rand. Init.	−	40.32	42.74	41.94	45.16	34.68	34.68	38.71	40.32	34.68	42.74	31.45	38.86
	CrossFormer	ZS	DenseGait	75.00	70.16	70.97	62.10	44.35	39.52	39.52	51.61	56.45	45.16	50.00	54.99
	CrossFormer	ZS	GREW	41.94	41.13	40.32	28.23	33.06	17.74	22.58	26.61	26.61	25.81	20.16	29.47
	CrossFormer	FT	DenseGait	59.68	70.16	70.16	66.13	56.45	54.84	49.19	56.45	59.68	62.90	48.39	59.46
	CrossFormer	FT	GREW	58.06	62.90	63.71	50.81	51.61	48.39	46.77	41.13	50.81	52.42	40.32	51.54

**Table 4:** Fine-grained comparison for each architecture on FVG, for all walking variations. For each model, we compared the performance between Zero-Shot (*ZS*) and Fine-Tuned (*FT*) for models pretrained on DenseGait and GREW, respectively, and no pretraining (*Rand. Init.*).

FVG								
**Architecture**	**Kind**	**Pretraining Dataset**	**WS**	**CB**	**CL**	**CBG**	**ALL**	**Mean**
SimpleViT	Rand. Init.	−	69.33	81.82	36.32	70.51	69.33	65.46
SimpleViT	ZS	DenseGait	71.00	57.58	43.16	70.09	71.00	62.57
SimpleViT	ZS	GREW	68.67	69.70	27.78	65.81	68.67	60.13
SimpleViT	FT	DenseGait	88.33	90.91	49.57	87.18	88.33	80.86
SimpleViT	FT	GREW	87.67	90.91	44.44	91.45	87.67	80.43
CaiT	Rand. Init.	−	57.67	72.73	25.21	65.81	57.67	55.82
CaiT	ZS	DenseGait	75.00	72.73	43.16	80.77	75.00	69.33
CaiT	ZS	GREW	66.33	54.55	32.48	68.80	66.33	57.70
CaiT	FT	DenseGait	86.00	90.91	45.73	86.32	86.00	78.99
CaiT	FT	GREW	82.00	78.79	45.30	82.05	82.00	74.03
Token2Token	Rand. Init.	−	66.33	81.82	32.48	72.65	66.33	63.92
Token2Token	ZS	DenseGait	73.33	63.64	43.16	81.20	73.33	66.93
Token2Token	ZS	GREW	53.00	57.58	24.79	58.55	53.00	49.38
Token2Token	FT	DenseGait	87.00	93.94	49.15	88.46	87.00	81.11
Token2Token	FT	GREW	82.33	78.79	34.62	84.62	82.33	72.54
Twins-SVT	Rand. Init.	−	30.67	48.48	16.67	32.05	30.67	31.71
Twins-SVT	ZS	DenseGait	70.33	60.61	42.74	78.21	70.33	64.44
Twins-SVT	ZS	GREW	61.00	66.67	25.21	53.85	61.00	53.55
Twins-SVT	FT	DenseGait	87.67	90.91	43.59	84.62	87.67	78.89
Twins-SVT	FT	GREW	85.33	69.70	33.76	84.62	85.33	71.75
CrossFormer	Rand. Init.	−	74.00	87.88	41.45	78.63	74.00	71.19
CrossFormer	ZS	DenseGait	77.67	72.73	45.30	79.49	77.67	70.57
CrossFormer	ZS	GREW	54.33	48.48	17.09	50.85	54.33	45.02
CrossFormer	FT	DenseGait	89.33	93.94	50.85	92.74	89.33	83.24
CrossFormer	FT	GREW	87.00	90.91	47.44	85.90	87.00	79.65

## Data Availability

The data used in this study are publicly available under open credentialed access. Request can be granted from the authors of the DenseGait, GREW, CASIA-B and FVG datasets.

## Abbreviations

The following abbreviations are used in this manuscript:

LN	LayerNorm
MSA	multi-head self-attention
FFN	feedforward network
SS	soft split
SSSA	spatially separable self-attention
LSA	locally-grouped self-attention
GSA	global sub-sampled attention
SDA	short-distance attention
LDA	long-distance attention
TSSI	tree skeleton structure image
SupCon	supervised contrastive
FVG	front-view gait
GREW	gait recognition in the wild
ViT	vision transformer
